# Comparative Genome Analysis of 16SrXII-A ‘*Candidatus* Phytoplasma solani’ POT Transmitted by *Hyalesthes obsoletus*

**DOI:** 10.3390/microorganisms14010226

**Published:** 2026-01-19

**Authors:** Anna-Marie Ilic, Natasha Witczak, Michael Maixner, Aline Koch, Sonja Dunemann, Bruno Huettel, Michael Kube

**Affiliations:** 1 Department of Integrative Infection Biology Crops-Livestock, University of Hohenheim, 70599 Stuttgart, Germany; 2 Institute for Plant Protection in Fruit Crops and Viticulture, Julius Kühn-Institute, Federal Research Institute for Cultivated Plants, 76833 Siebeldingen, Germany; natasha.witczak@julius-kuehn.de (N.W.); michael.maixner@julius-kuehn.de (M.M.); 3 Institute of Plant Science, Plant RNA Transport, University of Regensburg, 93053 Regensburg, Germany; aline.koch@ur.de; 4 Max Planck-Genome-Centre Cologne, 50829 Cologne, Germany; sdunemann@mpipz.mpg.de (S.D.); huettel@mpipz.mpg.de (B.H.)

**Keywords:** plant pathogenic bacteria, stolbur, effectors, virulence, potato

## Abstract

‘*Candidatus* Phytoplasma solani’ of the 16SrXII group is an emerging vector-borne pathogen in European crop production. The cixiid planthopper *Hyalesthes obsoletus* transmits 16SrXII-A stolbur phytoplasmas that are associated with diseases in grapevine, potato, and various weeds. While 16SrXII-P genomes transmitted by *Pentastiridius leporinus* are available, no genome of an *H. obsoletus*-transmissible 16SrXII-A phytoplasma has been reported from Germany. Here, we present insights into the phylogenetic position and pathogen–host interactions through the functional reconstruction of the complete 832,614 bp genome of the *H. obsoletus* transmissible ‘*Ca*. P. solani’ 16SrXII-A strain POT from a potato field. Phylogenetic analyses highlight the heterogeneity within the stolbur group using whole-genome alignment and a BUSCO-based core gene analysis approach. The POT chromosome shares highest average nucleotide identity with Italian bindweed-associated genomes and displays strong synteny with the c5 strain. Consistent with the typical phytoplasma architecture, the POT genome combines mobile-element-driven instability with a conserved core metabolism. Virulence factors include transposon-linked effectors but lack pathogenicity island organisation. POT further differs from other 16SrXII-group phytoplasmas through unique collagen-like proteins that could contribute to virulence. These findings provide a robust genomic framework that improves diagnostics, enables strain-level resolution and supports the assessment of breeding materials under stolbur phytoplasma pressure, thereby refining our understanding of stolbur phytoplasma diversity and highlighting the evolutionary divergence within the 16SrXII subgroup.

## 1. Introduction

Phytoplasmas are cell wall-less, phloem-limited bacteria of the class *Mollicutes*, exhibiting obligate biotroph in plant hosts and their hemipteran insect vectors [1,2]. With a broad host range and complex vector relationships, these pathogens pose a serious threat to agricultural production, and no curative control is currently available [3]. Their genomes are small, highly A + T-rich, shaped by regressive evolution and extensive gene loss, reflecting profound metabolic dependency on their hosts, while remaining paralog-rich and exhibiting remarkable genomic plasticity [4,5,6]. Complex transposons with characteristic gene content (potential mobile units) and phage-derived sequences, contribute to their dynamic genome structure [4,6,7]. Due to extensive genome reduction and dependency on host-derived nutrients, phytoplasmas remain uncultivable in axenic media—a limitation that continues to constrain functional and phenotypic characterization [6,7]. Species delineation and taxonomic classification within the provisional genus ‘*Candidatus* Phytoplasma’ relies on marker genes [8,9,10]. *16S rRNA* gene is particularly important, as its restriction fragment length polymorphism (RFLP) analysis enables assignment to ribosomal groups (16Sr groups) and their associated subgroups [9].

In Europe, phytoplasmas of the 16SrXII ribosomal group, particularly ‘*Candidatus* Phytoplasma solani’ (stolbur phytoplasma), have become increasingly relevant pathogens in major cropping systems. The disease was first identified in potatoes and tomatoes in southern and eastern Europe, as well as in peppers in southern Russia [11,12]. In the 1980s, a yellowing disease appeared in lavender (*Lavandula angustifolia* Mill.) and lavandin (*L.* x *intermedia* Loisel.) in France. This disease was also classified as stolbur due to the presence of the known stolbur-vector, *Hyalesthes obsoletus* Signoret [13]. In 1961, the grapevine disease ‘*bois noir*’ (black wood disease) was recognized as a distinct disease in north-eastern France when it was determined that it was not spread by the vector *Scaphoideus titanus* Ball. Today, ‘*bois noir*’ is known to be widespread and one of the most significant diseases in viticulture [14,15]. Later, it was discovered that the yellowing diseases affecting solanaceous plants, grapevines, lavender and other wild and cultivated plants were caused by a single species, ‘*Ca.* P. solani’.

Despite different vector systems, preferred host plants and high genetic variability, the strains are classified as ‘*Ca.* P. solani’ and, together with other phytoplasma species, form the 16SrXII group of stolbur pathogens [16]. This phylogenetic branch includes the recently emerged 16SrXII-P subgroup, first detected in sugar beet (*Beta vulgaris* L. subsp. *vulgaris*) in 2022, and has since spread rapidly across Germany [17].

In sugar beet, 16SrXII-P is transmitted primarily by *Pentastiridius leporinus* L., although *H. obsoletus* is also been present in sugar beet fields [18]. Experimental transmission assays showed that *H. obsoletus* populations associated with bindweed were able to transmit stolbur phytoplasmas to sugar beet, whereas nettle-associated populations were not [19]. 16SrXII-P represents a distinct lineage, separated from classical stolbur pathogens in potatoes (*Solanum tuberosum* L.) and other 16SrXII-A strains [20]. The latter is transmitted primarily by *H. obsoletus* [21,22], which is widely distributed in Central Europe and has been confirmed as a major vector of ‘*Ca.* P. solani’ to grapevine [23], potato [24], and other crops [19]. As disease occurrence is driven more by the distribution of reservoir weeds than by vector density [25], the ecology of *H. obsoletus* is central to stolbur epidemiology. Nymphs overwinter on the roots of infected weed hosts [26] and adults transmit the pathogen at high efficiency [27]. The interaction between vectors, reservoirs, and crops highlights the vulnerability not only of grapevines but also of other hosts, including solanaceous plants.

In potato, phytoplasmas from nine ribosomal groups have been detected (16SrI, 16SrII, 16SrIII, 16SrV, 16SrVI, 16SrVIII, 16SrXII, 16SrXIII, 16SrXVIII), with the 16SrXII-A subgroup most frequently reported across European potato production [24,28,29,30,31]. Stolbur in potatoes can cause yield losses of 30–80% and significantly reduce the quality of seed potatoes in Europe [32,33]. This severely limits their use in the food industry and for producing potato starch. In infested regions, producing planting material is virtually impossible as infected tubers either hamper in germination or do produce thread-like sprouts. Potatoes are a versatile raw material used in food production and agriculture. In 2023, Germany produced approximately 11.6 million tonnes of potatoes, accounting for nearly one-quarter of the European Union’s total harvest [34]. The ongoing spread of the vector and associated infections threatens potato cultivation in Germany’s agricultural landscape. Given their economic impact, genomic insights into phytoplasma–potato interaction are of particular importance since no resistant varieties are available.

Here, we present the comparative analysis of the complete genome sequence of strain POT, a 16SrXII-A subgroup member of ‘*Ca.* P. solani’, isolated from the experimental host *Catharanthus roseus* L. after transmission by the cixiid *H. obsoletus*, which was collected from potato field. Comparative analyses of POT with other 16SrXII phytoplasmas provide new insights into the diversity and pathogen–host interaction, improving our understanding of stolbur.

## 2. Materials and Methods

*H. obsoletus* individuals were collected from a symptomatic potato field in Bingen (Rhineland-Palatinate, Germany; 49.952973° N, 7.927214° E) and used for experimental transmission to *C. roseus* plants in a no-choice setup under controlled conditions. *C. roseus* plants (derived from vegetative propagation) were cultivated in 2 L pots containing pathogen-free soil and maintained under insect-poof greenhouse conditions at the Julius Kühn-Institute, Siebeldingen (Germany). Environmental conditions set to 25 ± 5 °C, 70 ± 10% relative humidity, a 16:8 light/dark photoperiod, and minimum light intensity of 2500 lx. Each *C. roseus* plant was placed in a cylindrical acrylic insect-proof cage [35] and hosted up to ten captured *H. obsoletus* individuals for an inoculation access period (IAP) of 10 days, with seven plants exposed and two serving as negative controls. Transmission experiments were conducted in a climatic insect chamber (Fitotron type SGR233, Weiss Technik Ltd., Loughborough, UK) under day/night temperatures of 23:18 ± 2 °C, 50 ± 5% relative humidity, and a 16:8 h photoperiod. Insect cages were inspected daily, and deceased insects were removed, stored at −20 °C, and retained for subsequent morphological identification and molecular analyses. After completion of the IAP, all plants were removed from the cages and treated repeatedly with insecticide (Spruzit Schädlingsfrei, W. Neudorff GmbH KG, Emmerthal, Germany) to eliminate remaining insects. Plants were then maintained under greenhouse conditions for ten weeks to allow symptom development. DNA was extracted from midrib tissue using solid-phase extraction with the NucleoBond HMW DNA Kit (Macherey-Nagel, Düren, Germany), following the manufacturer’s protocol for plant material. The DNA concentration was quantified using a Qubit fluorometer (Thermo Fisher Scientific, Waltham, MA, USA). Phytoplasma infection was confirmed by endpoint PCR using the universal primer pairs P1/P7 [36,37] and R16F2n/R2 [38], both targeting regions of the rRNA operons, and further validated by sequencing of the PCR products.

A whole genome shotgun library for single-molecule real-time (SMRT) sequencing was prepared with the SMRTbell prep kit v3.0, SPK3.0 (Pacific Biosciences, Menlo Park, CA, USA) without additional DNA fragmentation. Short fragments were removed using 35% diluted PacBio AMPure PB beads, following the low-input protocol (Pacific Biosciences, Menlo Park, CA, USA). Library sequencing was performed on a Revio device with a 25M ZMW SMRT cell and SPRQ sequencing chemistry and SPRQ polymerase at the Max Planck Genome-centre Cologne. This was followed by the quality filtering and processing of the raw reads using the SMRTlink suite version 25.1.0.257715 including adapter trimming.

Taxonomic binning was then performed using DIAMOND v0.9.30.131 [39] with the option BLASTX against the NCBI nonredundant protein database (downloaded on 10 December 2024) and MEGAN v7.1.0 [40]. Long reads (>10 kb) assigned to the taxonomic rank ‘*Candidatus* Phytoplasma’ were selected for genome assembly using Canu v2.2 [41] with a pacbio-hifi option and an estimated genome size of 1.1 Mb. Genome circularity was indicated by the assembler. However, the consensus contig retained a terminal end-to-end overlap, as expected for circular contigs assembled by Canu v2.2 [41]. The trimming points reported by the assembler were verified by BLASTN v2.11.0 [42] and the redundant terminal region was removed in Artemis v18.2.0 [43], yielding a contiguous, circular sequence. Subsequently, the sequence was reoriented to start at the *dnaA* gene based on the cumulative GC-skew [(G − C)/(G + C)] analysis. The use of *dnaA* follows a common convention for circular bacterial genomes, as it encodes the replication initiator protein DnaA that triggers chromosomal DNA replication and is typically located close to the origin of replication [44,45]. Long-read mapping using pbmm2 v1.17.0 (https://github.com/PacificBiosciences/pbmm2 (accessed on 30 June 2025)) was performed to validate assembly accuracy and coverage uniformity, and CheckM2 v1.1.0 [46] was used to independently estimate genome completeness and potential contamination based on conserved marker genes. Assembly quality was further assessed using Benchmarking Universal Single-Copy Orthologs (BUSCO) v5.8.1 [47] with 151 single-copy orthologs from the class *Mollicutes*.

Initial annotation was done automatically by the Rapid Annotation using Subsystem Technology (RAST) v2.0 pipeline [48], with parameters set to translation table 11 and the NCBI taxonomy ID: 69896 for ‘*Ca*. P. solani’. The result was refined in Artemis v18.2.0 [43], incorporating NCBI Prokaryotic Genome Annotation Pipeline (PGAP) v.6.10 [49] and BLASTP v2.11.0 [42] comparison of the deduced protein sequences against the non-redundant protein database (accessed on 12 December 2024) for refinement. Metabolic pathway reconstruction and functional annotation were performed using BlastKOALA v3.1 [50] and InterProScan v.106.0 [51], while membrane and secreted proteins were predicted with Phobius v1.01 and aligned across complete phytoplasma genomes using BLASTPv2.11.0 and TBLASTN v2.11.0 [52]. To confirm the taxonomic placement of the organism, a BLASTN v2.11.0 [42] analysis of the *16S rRNA* gene sequence was performed, followed by assignment using iPhyClassifier [53]. The PacBio SMRT sequencing reads used for genome assembly in this study have been deposited in the Sequence Read Archive (SRA) under accession number SRR35343499 (BioProject PRJNA1327367, BioSample SAMN51278244). Sequence and annotation of strain POT has been deposited in GenBank under accession number CM135798.1.

For comparative analysis, the newly assembled POT genome was analysed alongside complete genome sequences from four 16SrXII-A strains—c1 (GenBank accession: CP103788), c4 (GenBank accession: CP103787), c5 (GenBank accession: CP103786), o3 (GenBank accession: CP103785) and two 16SrXII-P strains, GOE (GenBank accession: CP155828) and PENLEP [54]. RNA features in the PENLEP genome were manually curated due to incomplete annotation, to ensure comparable genomic benchmarks across strains.

To ensure comparability of *16S rRNA* gene analyses, all *16S rRNA* gene sequences were aligned using MAFFT v7.505 [55], and consistently trimmed to identical homologous regions defined by conserved multiple sequence alignment coordinates. Pairwise sequence identity of the *16S rRNA* gene was calculated using BLASTN v2.11.0 [42], while whole-genome similarity was evaluated based on Average Nucleotide Identity (ANI) using FastANI v1.34 [56]. In addition, genome organization was compared by whole-genome alignment using Mauve v2.4.0 [57]. For the comparison of shared and unique genomic features among the complete 16SrXII stolbur phytoplasma genomes, orthogroups of the predicted amino acid sequences were identified using OrthoFinder v2.5.5 [58]. Phylogenomic analysis was performed using the core set of complete single-copy orthologs identified by BUSCO v5.8.1 [47], which incorporates Prokaryotic Dynamic Programming Genefinding Algorithm (Prodigal) [59] for standardized protein prediction. Complete phytoplasma genomes (Supplementary Table S1) were retrieved from the NCBI Genome Data Set on 15 July 2025. Sequence similarity was assessed using BLASTP v2.11.0 [42] to provide pairwise identity matrix for amino acid-level comparison, providing a metric for distinguishing closely related species. Multiple sequence alignment was performed using MAFFT v7.505 [55]. Phylogenetic reconstruction was conducted using the maximum likelihood method with 1000 bootstrap replicates implemented in IQ-TREE v2.4.0 [60]. The resulting phylogenetic tree was visualized using Molecular Evolutionary Genetics Analysis (MEGA) v12.0.11 [61]. In addition, phylogenetic reconstruction was also performed on selected near-full length *16S rRNA* sequences from reference taxa [10], which were trimmed to identical start (5′-TGCAAGTCGAAC-3′) and end positions (5′-GAAGGTRGGGAT-3′) to ensure comparability. Since the reference sequence of ‘*Ca.* P. australiense’ was too short, strain PAa (GenBank accession: AM422018) was used instead. Unless otherwise stated, default parameters were applied.

## 3. Results

### 3.1. Transmission to Non-Infected C. roseus Plants

**Figure 1:**
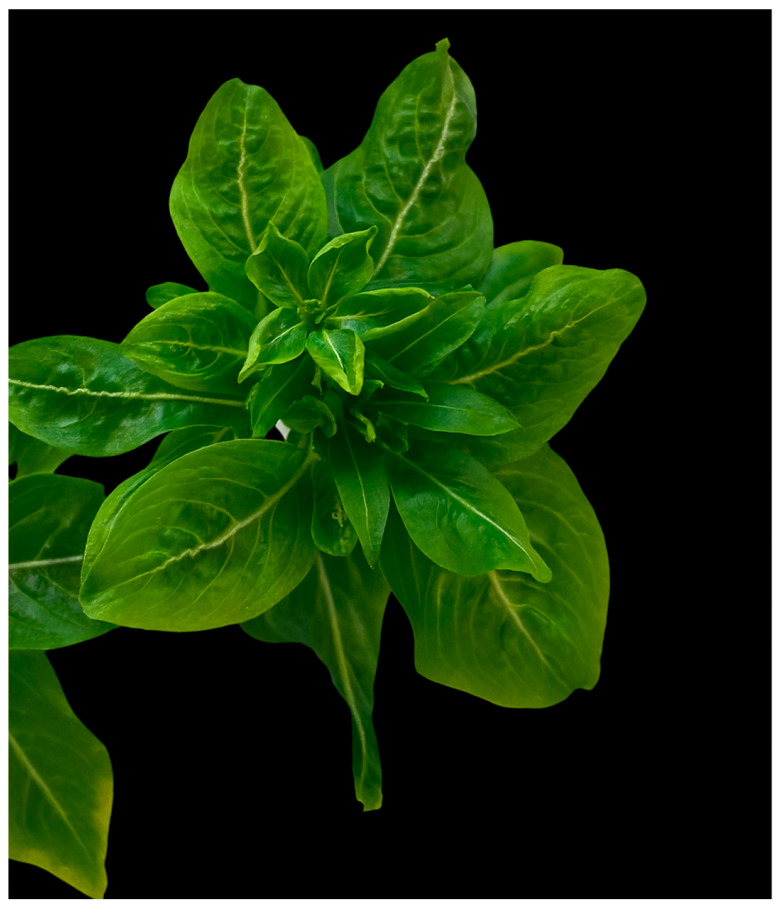
Virescence associated with ‘*Ca*. P. solani’ POT infection in *C. roseus*.

Transmission success was assessed based on infection rates in recipient plants, with all negative control plants remaining phytoplasma-negative throughout the experiment. Among the seven *C. roseus* plants exposed to *H. obsoletus*, five plants (71.43%) tested positive for phytoplasma infection. Infected *C. roseus* plants exhibited typical phytoplasmoses symptoms, including stunting, shoot proliferation, leaf crinkling, phyllody, and virescence. (Figure 1). Regarding the insects used for transmission, of the 56 individuals initially used, 48 were recovered after the inoculation period, and 13 of these (27.08%) tested positive for phytoplasma.

### 3.2. Genomic Benchmarks of Complete 16SrXII-A and -P Genomes

**Figure 2:**
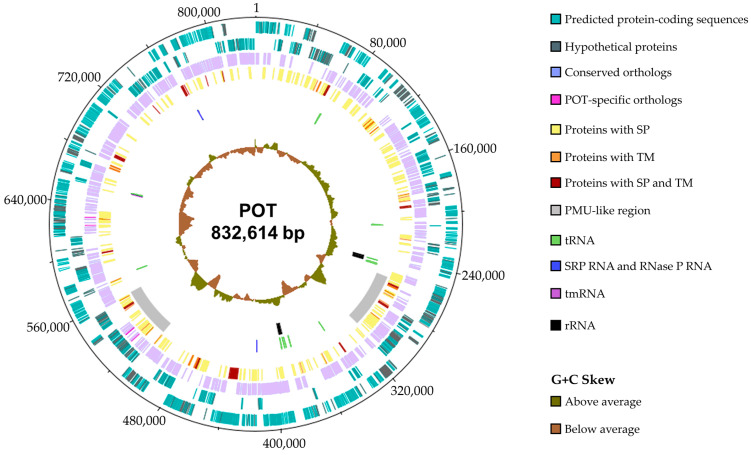
Genome visualization of the circular chromosome of strain POT. From the outermost to the innermost ring: the chromosomal coordinate scale (in bp); predicted protein-coding sequences and hypothetical proteins, shown as separate tracks for the forward and reverse strands; conserved orthologs shared with all analysed ‘*Ca*. P. solani’ genomes and POT-specific orthologs. Predicted membrane proteins are categorised by structural features, including proteins with a signal peptide (SP), proteins with transmembrane domains (TMs), and proteins containing both a SP and TMs. PMU-like regions and RNA elements are indicated, including signal recognition particle (SRP) RNA and RNase P RNA component class B, transfer RNAs (tRNAs), transfer-messenger RNAs (tmRNAs), and rRNAs. The innermost ring represents the G + C skew.

SMRT sequencing resulted in a total of 1,929,813 long reads. Subsequent taxonomic binning assigned 316,493 reads (N_50_: 7.26 kb) to the genus ‘*Candidatus* Phytoplasma’. For genome assembly, a subset of 60,609 reads exceeding 10 kb in length (N_50_: 13.91 kb) was selected, resulting in a single circular contig of 832,614 bp with a G + C content of 28.21% and an average assembly coverage of 245.67-fold (Figure 2). Circularity of the assembled genome was confirmed by the presence of a terminal overlap exceeding 22 kb. The genome was uniformly covered across its entire length, with 100% of bases supported by mapped reads and an average read-mapping depth of approximately 1010×, and no evidence of low-coverage regions. The difference between assembly coverage and read-mapping depth reflects the effective read subset used during genome assembly.

Assembly completeness and potential host admixture were evaluated using CheckM, which estimated a completeness of 99.45% and a contamination level of 0.43%, supporting the absence of significant non-phytoplasma sequences after binning. Assembly completeness was further assessed using BUSCO, revealing 95.3% completeness, comprising 94.0% single-copy and 1.3% duplicated BUSCOs, comparable to other analysed 16SrXII phytoplasma genomes [62] and showing no increase in fragmented or missing BUSCOs. A duplication of DNA helicase was observed across all analysed genomes, while an additional duplication of *dnaG* primase was detected in POT.

In accordance with previously published complete genomes of the taxon ‘*Ca.* P. solani’, this assembly did not yield any plasmids and confirmed the circular chromosome architecture. The POT genome shares (Table 1) typical benchmarks of phytoplasmas [6], including two ribosomal RNA (rRNA) operons, 32 transfer RNA (tRNA) genes, two ncRNAs and a single transfer-messenger RNA (tmRNA). Strains from subgroup 16SrXII-P (GOE and PENLEP) exhibit consistently lower G + C content (~26.2%) and condensed chromosomes (~700 kb) compared to subgroup A strains, which reach up to 28.58% G + C content and nearly 974 kb in chromosome length. Differences in coding density and average CDS length between 16SrXII-A and 16SrXII-P are subtle, ranging from 0.891 to 1.027 CDS per kb and from 0.723 to 0.864 kb, respectively. In contrast, the total number of CDS differs more clearly, with 16SrXII-P encoding between 637 and 663 CDS and 16SrXII-A encoding between 719 and 1000 CDS. Notably, strain o3 exhibits the longest chromosomal length, approximately 973,640 base pairs, the highest CDS count of 1000, and the greatest coding density of 1.027 CDS per kilobase within the dataset. Across all strains, 15–50% of CDS are annotated as hypothetical proteins, underscoring the large proportion of uncharacterized gene content typical for phytoplasmas.

### 3.3. Comparative Genome Analyses

#### 3.3.1. Genome Organization Analysis

**Figure 3:**
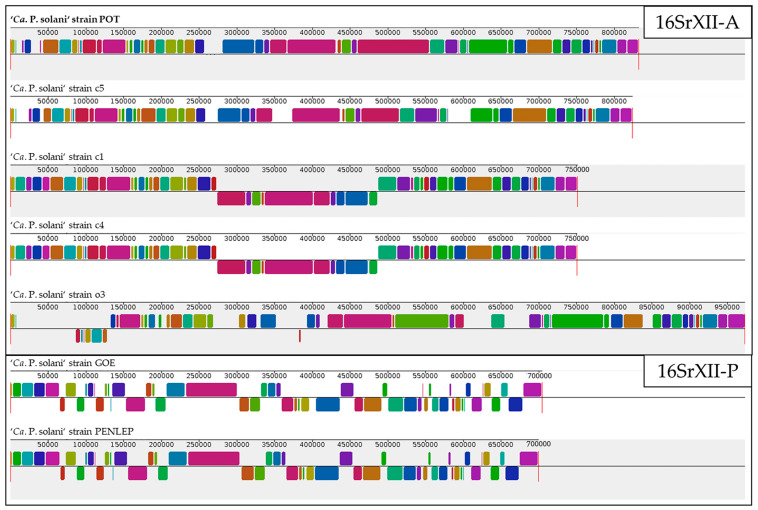
Alignment of the genome sequences from seven complete stolbur phytoplasma strains. Colored blocks represent LCBs identified by Mauve, with identical colors indicating conserved syntenic regions across genomes. Numbers along the horizontal axis indicate genome positions (bp). Block orientation is shown relative to strain POT, with inversions indicated by blocks plotted below the central axis. Strain POT is highlighted in bold.

In the Mauve whole-genome alignment of ‘*Ca.* P. solani’, strain o3 stands out as the most structurally divergent genome across all comparisons (Figure 3). Within the 16SrXII-A group, the stinging nettle-originated strain o3 shows the weakest collinearity, with the largest number of locally collinear block (LCB) breakpoints, multiple long inversions, and several lineage-specific blocks that are either truncated or expanded relative to the other strains. In contrast, POT exhibits a strong level of collinearity within the bindweed-subgroup, showing the strongest synteny with c5. Both share extensive LCBs, with only minor differences in block size interrupting an otherwise highly conserved backbone. These LCBs encompass the majority of predicted coding sequences, indicating conservation of gene order and genomic context across the compared strains. When compared to other 16SrXII-A strains (c1, c4, and o3), POT still retains the characteristic genomic framework of this lineage, but the alignment reveals more substantial rearrangements and inversions in these additional strains. The comparison with the 16SrXII-P representatives GOE and PENLEP underscores the clear genomic distinction between the A- and P-type strains [20], supported by reduced synteny and extensive block rearrangement relative to the A-type.

#### 3.3.2. Assessing Genomic Relatedness Using ANI and *16S rRNA* Similarity

**Figure 4:**
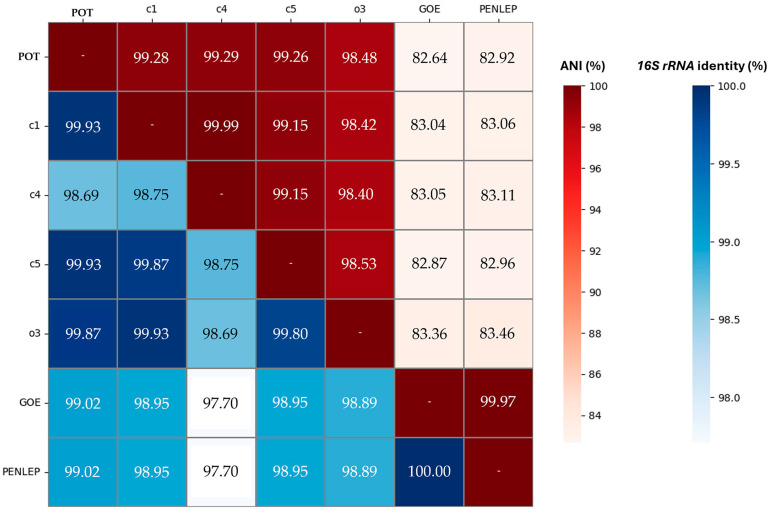
Heatmap of pairwise ANI and *16S rRNA* gene sequence identity among seven complete stolbur phytoplasma genomes. The heatmap displays pairwise ANI values (upper right triangle, red shading) and *16S rRNA* gene sequence identities (lower left triangle, blue shading), with colour bars indicating the percentage identity for each metric.

To determine the taxonomic assignment of the phytoplasma, the full-length *16S rRNA* gene sequence was analysed using iPhyClassifier. The query sequence (PSOL_02220) shared 99.67% nucleotide identity with the reference strain of ‘*Ca.* P. solani’ (GenBank accession: AF248959), placing the strain within the ‘*Ca*. P. solani’ taxon. Moreover, the newly identified strain POT shows a high genomic similarity to available ‘*Ca*. P. solani’ strains. ANI values between POT and strains c1, c4, c5, and o3 exceed 98% (Figure 4), ranging from 98.48% to 99.93%. The *16S rRNA* gene sequence identity between POT and these strains consistently exceeds 99%, which further corroborates this taxonomic placement. In contrast, the strains GOE and PENLEP, both assigned to the 16SrXII-P subgroup, display significantly lower ANI values when compared to POT and other ‘*Ca*. P. solani’ strains, with values around 82% to 83%. These low ANI values clearly delineate the 16SrXII-P subgroup as a separate species lineage from ‘*Ca*. P. solani’ according to current taxonomic standards [20]. Notably, PENLEP and GOE share a 100% identical *16S rRNA* gene sequence and an exceptionally high ANI value of 99.97%, indicating a nearly identical genome. Despite their high intra-group similarity, their genomic distance to the ‘*Ca*. P. solani’ cluster remains substantial.

#### 3.3.3. Phylogenomic Positioning of Stolbur Phytoplasmas

**Figure 5:**
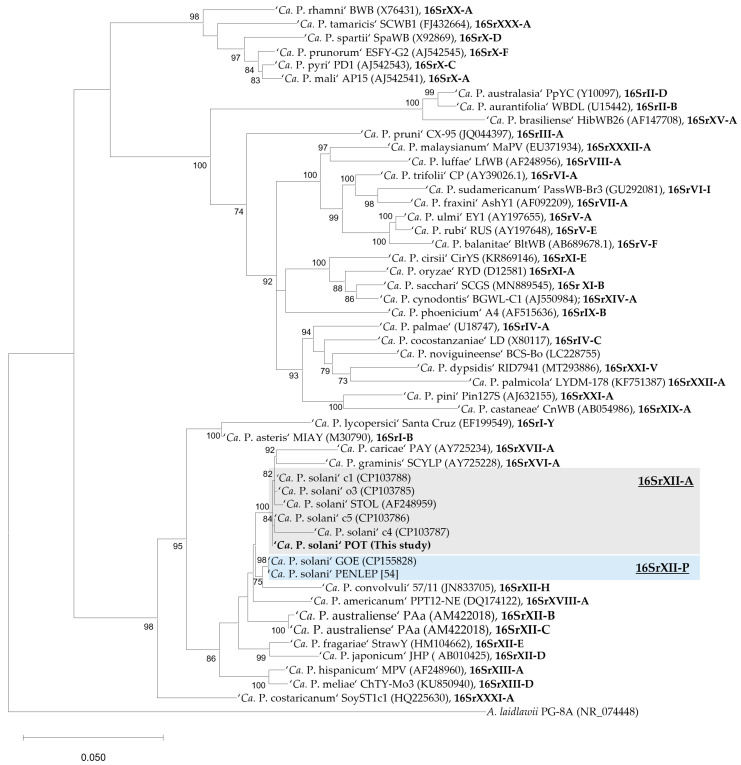
Maximum-likelihood phylogeny of 44 selected reference phytoplasma taxa and the seven complete stolbur genomes based on near-full length *16S rRNA* gene (1549 aligned nucleotide positions, 553 variable sites), with *Acholeplasma laidlawii* PG-8A as outgroup. Maximum-likelihood inference was performed under the TIM2+F+R3 model, selected as the best-fit model based on the Bayesian Information Criterion (BIC). The final tree has a log-likelihood of –10,608.217699. Only bootstrap support values ≥ 70 are shown. Scale bar indicates substitutions per site. Strain POT is highlighted in bold. Where available, 16Sr group assignment is indicated in bold.

In *16S rRNA* analysis, strain POT is assigned to 16SrXII-A subgroup. In the maximum-likelihood tree based on nearly full-length *16S rRNA* sequences (Figure 5), the P-subgroup strains are positioned distinctly apart from the reference strain STOL and the A-type cluster. Instead, they form a separate, well-supported branch (bootstrap = 99) that groups more closely with the 16SrXII-H type strain ‘*Ca.* P. convolvuli’. In contrast, strain POT is placed firmly within the A-type lineage of ‘*Ca.* P. solani’, close to the strains c1, c4, c5, o3, and STOL, a grouping captured with high bootstrap support. Within the A-type clade, the nettle-derived strain o3 did not segregate from the other A-type strains, which is consistent with their high *16S rRNA* sequence identity (Figure 4).

**Figure 6:**
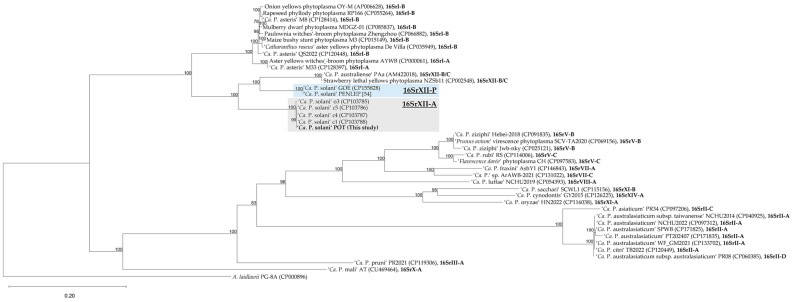
Maximum-likelihood phylogeny of 40 complete ‘*Candidatus* Phytoplasma’ genomes based on a core SCO set, with *A. laidlawii* PG-8A as outgroup. The phylogenetic reconstruction is based on an alignment of a concatemer of 41 SCOs identified with BUSCO, comprising 12,956 aligned aa positions with 8206 variable sites. Maximum-likelihood inference was performed under the JTTDCMut+F+I+R4 model, selected as the best-fit model based on the Bayesian Information Criterion (BIC). The final tree has a log-likelihood of −197,743.1000. Only bootstrap support values ≥ 70 are shown. Scale bar indicates substitutions per site. Strain POT, along with the respective 16Sr group assignments for each strain, are highlighted in bold.

Because the *16S rRNA* gene is limited by functional constraints and prone to genetic saturation, we performed a phylogenomic analysis (Figure 6) to provide a more robust framework for subgroup assignment. The evolutionary relationships within the genus were inferred from a concatenated alignment of 41 universally conserved single-copy orthologs (Supplementary Table S2), identified using mollicutes-specific BUSCO marker set, which enables reliable inference. The resulting amino acid alignment comprised 12,956 aligned aa positions of which 8206 were parsimony-informative sites—indicating substantial phylogenetic signal and high resolution for delineating evolutionary relationships within phytoplasma lineages. The resulting maximum likelihood tree (Figure 6) resolves the genus into two major monophyletic clades with strong bootstrap support (≥83 across all internal nodes). The first clade comprises representatives of the 16SrXII and 16SrI subgroups, while the second encompasses the 16SrII, 16SrV, 16SrX, and 16SrXIII subgroups. Within the 16SrXII clade, the phylogenomic analysis reveals clear subclade differentiation. Strains assigned to the provisional P-type, including PENLEP and GOE, form a strongly supported cluster (bootstrap = 100) that is positioned closer to the B/C-type cluster than to A-type strains. In contrast, strain POT clustered within the 16SrXII-A clade, grouping together with the strains c1, c4 and c5.

Moreover, this approach confirmed distinct position of the nettle-originated strain o3, which was separated from the remaining A-type strains with high bootstrap support (=100%). This finding is consistent with ANI results (Figure 4) and provides a resolution that could not be achieved using *16S rRNA*-based analysis (Figure 4 and Figure 5).

To assess genome-wide similarity and species boundaries among all currently available complete phytoplasmas genomes, a core BUSCO-based identity metric (cBUSCO) was calculated as the mean pairwise amino acid identity across 41 universal single-copy BUSCO markers (Supplementary Table S2). Using BLASTP v2.11.0, we observed a separation of cBUSCO identity values around 97.8%, which was therefore used as a tentative reference threshold. The matrix (Table 2) shows that strains within 16SrXII-A share cBUSCO identity values above the 97.8% threshold (≥99.76%), while identities between members of 16SrXII-A and the 16SrXII-P or 16SrXII-B/C subgroups drop sharply to ~84–85%. These values fall far below 97.8%, supporting a separation consistent with distinct species-level lineages [63]. In contrast, within-group comparisons for 16SrXII-P and 16SrXII-B/C also show high internal consistency (≥99.99% and ≥99.73%, respectively), further supporting the robustness of the cBUSCO threshold across the clade. Importantly, the cBUSCO-based species assignments are congruent with genome-wide ANI values (Figure 4). The use of the 97.8% cBUSCO threshold is further exemplified within the 16SrI lineage (Supplementary Table S3). The strains AYWB and M33, which form a well-supported clade in the phylogeny (Figure 6), share a high cBUSCO identity of 99.81%, placing them within the same species. In contrast, the remaining 16SrI strains (De Villa MBSP-M3, MDGZ-01, M8, OY-M, PaWB, QS2022, RP166) share values below 97.8% when compared to AYWB or M33, consistent with their phylogenetic separation reported previously using whole-genome ANI and multilocus markers [64]. Similarly, ‘*Ca*. P. rubi’ RS and ‘*Flavescence dorée*’ phytoplasma CH have a cBUSCO identity of only 96.69%, which places them below the species threshold for this characteristic and aligns with their current taxonomic classification. Conversely, ‘*Prunus avium*’ virescence phytoplasma SCV-TA2020 and the ‘*Ca*. P. ziziphi’ strains Hebei-208 and Jwb-nky share identities of at least 97.86%, with SCV-TA2020 and Jwb-nky being identical in their full-length *16S rRNA* gene, supporting their assignment to the same species [65].

### 3.4. Pan-Genome Analysis

Comparative orthogroup analysis identified 792 orthogroups across 5290 deduced amino acid sequences of the seven phytoplasma genomes analyzed. Of these, 5112 sequences (96.66%) were assigned to orthogroups, whereas 178 remained unassigned. In total, 31 species-specific orthogroups were detected, encompassing 98 sequences (1.92%). Of the 792 orthogroups, 403 were present in all genomes, with 358 representing single-copy orthogroups that define a robust core genome. Pairwise comparisons (Table 3) show that POT shares the highest number of orthogroups with c5 (541). Slightly fewer orthogroups are shared with c4 (537), c1 (534), and o3 (523), while it shares considerably fewer with the P-type strains PENLEP (434) and GOE (431).

Multi-copy genes were analysed to assess whether genome expansion in stolbur phytoplasmas correlates with an increased number of gene copies (Table 4). While c5 and POT share high identity values and exhibit high synteny in genome organization, the markedly higher number of multi-copy genes in POT could provide an explanation for its larger genome size. In c5, 214 multi-copy genes comprise 26.52% of all CDS and 17.11% of the chromosome, whereas 235 in POT constitute 31.76% and 20.77%, respectively, suggesting that even small copy number differences drive genome expansion.

In comparison, the smaller A-subgroup strains c1 and c4 encode only 135 (18.78%) and 139 (19.20%) multi-copy genes, both covering roughly 12% of their chromosomes, while o3 represents the extreme case with 413 multi-copy genes, accounting for 41.30% of all CDS and nearly one third of the chromosome. Within the P-subgroup, GOE and PENLEP show intermediate values, with 157 (23.68%) and 154 (24.18%) multi-copy genes, both occupying ~13% of their genome length. The variation in genome size among stolbur phytoplasma subgroups is closely linked to the abundance of multi-copy genes, a pattern also observed in members of the asteris group [64]. Beyond the quantitative differences, the functional composition of the multi-copy gene fraction provides further insight. Many of these copies correspond to transposon-related genes, such as *tra5*, *tmk*, *himA*, *sigB*, *ltrA*, *dnaB*, *dnaG*, *ssb*, and *ftsH*, along with SVM-like genes, while a considerable number represent hypothetical proteins.

**Figure 7:**
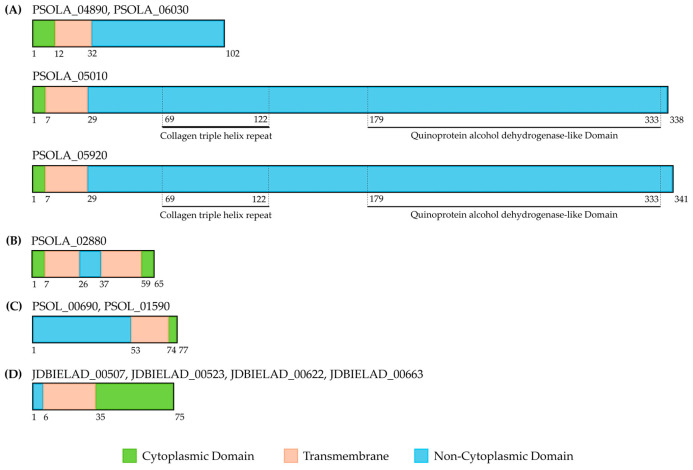
Structural characteristics of unique and shared orthologous proteins identified by OrthoFinder. (**A**) POT-specific orthologs; (**B**) ortholog shared between POT and GOE; (**C**) GOE-specific ortholog; (**D**) PENLEP-specific ortholog. Protein models are shown to scale in amino acids with locus tags and amino acid lengths. Cytoplasmic (green), transmembrane (orange), non-cytoplasmic (blue) regions, along with representative domains (highlighted with dashed lines and labelled) were predicted with InterProScan and are indicated with their respective positions.

Strain-specific orthogroups further highlight diversification within the A-subgroup: c5 carries seven unique orthogroups, o3 exhibits pronounced distinctiveness with 17 unique orthogroups, whereas c1 and c4 lack any unique orthogroup. POT comprises two exclusive orthogroups absent from all other genomes. The first POT-specific orthogroup encodes four proteins (PSOLA_04890, PSOLA_06030, PSOLA_05010, PSOLA_05920) with a conserved architecture including an N-terminal cytoplasmic domain (CD), a single transmembrane helix (TM), and a C-terminal extracellular domain (ECD) containing a predicted coiled-coil region (Figure 7A). BLASTP searches revealed ~50% sequence identity over 90 aa with a hypothetical protein (protein ID: MFG6084861.1) from ‘*Ca*. P. solani’ strain STOL. InterProScan indicated within the ECD a domain homologous to the influenza hemagglutinin stalk, a conserved α-helical element mediating membrane fusion in viruses [66], suggesting a potentially similar coiled-coil architecture that could facilitate membrane interactions in POT. The second POT-specific orthogroup encodes collagen-like proteins with Gly–X–Y repeats forming a triple helix (Figure 7A). InterProScan assigned a “quinoprotein alcohol dehydrogenase-like” annotation based on structural similarity within the ECD. This likely reflects fold rather than function, as phytoplasmas lack pyrroloquinole quinone (PQQ) pathways and such proteins likely mediate host adhesion [67]. BLASTP v2.11.0 analysis revealed that these proteins share a sequence identity of 78% over 316 aa with the hypothetical protein PSSA1_v1c4250 (protein ID: RMI88696.1) from ‘*Ca*. P. solani’ strain SA-1. However, InterProScan did not assign any functional annotation related to quinoprotein alcohol dehydrogenase-like proteins for this sequence.

Here, one orthogroup is shared exclusively between POT and GOE, while it was entirely absent from c1, c4, c5, o3 and PENLEP. The POT-encoded protein within this orthogroup, PSOLA_02880, is a small transmembrane protein predicted to contain two transmembrane helices (Figure 7B). Notably, it shows no detectable homology to any known protein family. BLAST analysis revealed a 100% sequence identity over the full length of 65 aa to the hypothetical protein PSSA1_v1c5470 (Protein ID: RMI87778.1) from ‘*Ca*. P. solani’ strain SA-1.

GOE harbours three unique orthogroups. The first two orthogroups, comprising the locus_tags PSOL_01770 and PSOL_06790, as well as PSOL_05410 and PSOL_06240, lack detectable protein domains or significant sequence similarity in BLAST searches. The third orthogroup, composed of PSOL_00690 and PSOL_01590 (Figure 7C), encodes a protein with an ECD-TM-CD topology, yet functional inference remains elusive. However, despite this structural organization, BLAST analysis again provided no further functional insight. PENLEP contains one unique orthogroup (Figure 7D; JDBIELAD_00507, JDBIELAD_00523, JDBIELAD_00622, and JDBIELAD_00663) without BLAST matches, and another (JDBIELAD_00072, JDBIELAD_00450) with partial similarity to an amino acid ABC transporter ATP-binding protein (AGL90923.1) from Strawberry lethal yellows phytoplasma NZSb11. Given the low similarities (34/243 and 77/243 aa) and short sequence lengths (44 aa and 88 aa), both open reading frames may encode non-functional remnants.

### 3.5. Functional Reconstruction

#### 3.5.1. Metabolic Pathways and Transportome

**Figure 8:**
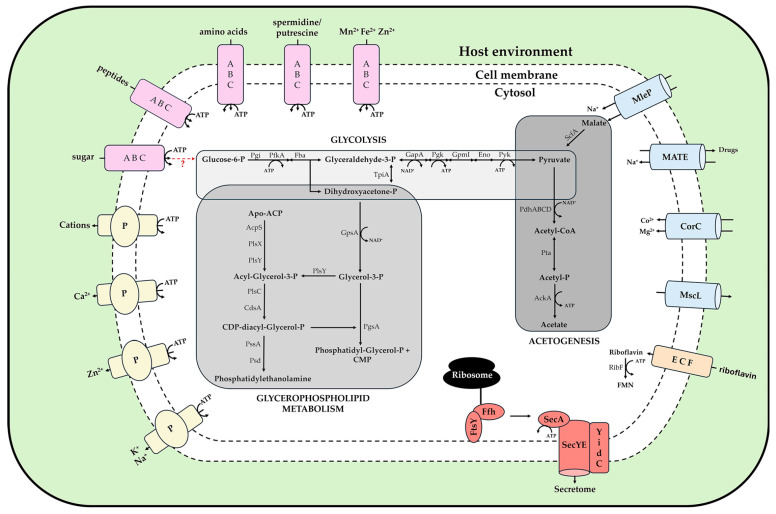
Schematic overview of complete metabolic repertoire and associated transport systems inferred from seven complete 16SrXII phytoplasma genomes. Elements involved in transports at the membranes (P-type (yellow), ABC transporters (purple), Sec-dependent secretion system (red), transport proteins (light blue) and riboflavin transporter (orange)) in ‘*Ca*. P. solani’ strains POT, c1, c4, c5, o3, GOE and PENLEP. Abbreviations for the depicted proteins and enzymes are stated in the results section of this study.

Consistent with the GOE genome reconstruction [20], comparative analysis of complete 16SrXII-A and 16SrXII-P type genomes reveals a conserved metabolic core (Figure 8). Central carbohydrate metabolism is limited to a near-complete glycolytic pathway from glucose-6-phosphate to pyruvate, catalysed by phosphoglucose isomerase (Pgi), phosphofructokinase (Pfk), fructose-bisphosphate aldolase (Fba), glyceraldehyde-3-phosphate dehydrogenase (Gap), phosphoglycerate kinase (Pgk), phosphoglycerate mutase (Pgm), enolase (Eno), and pyruvate kinase (Pyk), with ATP generated at the Pgk and Pyk steps. Pyruvate oxidation proceeds via the pyruvate dehydrogenase complex (PdhABCD), followed by acetogenesis through phosphotransacetylase (Pta) and acetate kinase (AckA), producing additional ATP. Malate uptake is mediated by MleP, with conversion to pyruvate via malate dehydrogenase (ScfA). Membrane lipid biosynthesis appears to follow the canonical PlsX–PlsY–PlsC–CdsA pathway, channelling acyl-acyl carrier protein intermediates into CDP-diacylglycerol for phosphatidylglycerol synthesis as a major structural glycerophospholipid. Phosphatidylethanolamine likely derives from CDP-diacylglycerol through the phosphatidylserine synthase (PssA)/phosphatidylserine decarboxylase (Psd) pathway [6]. Riboflavin is imported through an ECF transporter and converted to FMN/FAD by RibF [6,7]. Among the stolbur genomes analysed, only o3 encodes a sucrose phosphorylase GtfA (pso3_02210), whereas a phosphoglucomutase (Pgm) is not predicted, a pattern already observed for ‘*Ca*. P. australiense’ PAa [68], a strain of subgroup 16SrXII-B/C; and likewise reported for 16SrV phytoplasmas [69].

**Figure 9:**
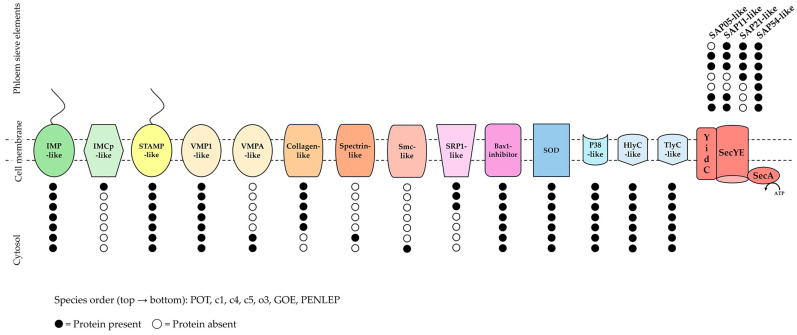
Schematic overview of putative virulence-associated proteins in stolbur phytoplasmas. For each protein, presence (black circle) or absence (white circle) of the protein is indicated across species. The species are arranged from top to bottom as follows: POT, c1, c4, c5, o3, GOE, PENLEP. Abbreviations for the depicted proteins are stated in the results section of this study.

The reduced metabolism is compensated by a broad set of transporters. The transportome includes multiple ABC systems for amino acids, polyamines, metal ions, peptides and sugars, as well as cation-transporting P-type ATPases, Na^+^/drug antiporters (MATE), cation diffusion facilitators (CorC) and mechanosensitive channels (MscL) [6,20]. Protein translocation is mediated by the sec-dependent secretion pathway, comprising SecYE, SecA and YidC, with FtsY and Ffh as components of the signal recognition particle apparatus [70]. Beyond the conserved central metabolic framework, the genomes share a stable core of putative effector genes, complemented by lineage-specific proteins that could influence host manipulation and pathogenicity (Figure 9).

#### 3.5.2. Membrane-Associated Surface Proteins

As wall-less plant pathogens, phytoplasmas are characterised by an innate, minimal yet specialised membrane architecture that underpins host interactions, immune evasion, and vector-mediated transmission. Among these, immunodominant proteins (IDPs) that are embedded in the cell membrane are suggested to play central roles in these processes [70].

All analysed genomes encode the set of IDPs (Figure 9), including immunodominant membrane protein (IMP), stolbur antigenic membrane protein (STAMP) and variable membrane protein 1 (VMP1) [20]. While STAMP occurs in all strains, a phytoplasma-specific AMP domain was detected by InterProScan only in the P-type strains GOE and PENLEP, indicating structural differences among lineages. This separation is further supported by pairwise BLASTP comparison of STAMP (Supplementary Table S4), which split GOE and PENLEP into a distinct P-type cluster (100% identity), clearly apart from the A-type genomes (≤41% identity to A-types). Within the A-type group, POT and c5 form a tight cluster (100% identity) as well as c1 and c4, while o3 is more divergent (~89–91% identity to other A-types), reflecting its peripheral position within the 16SrXII-A subgroup. In addition to the conserved IDPs, only POT encodes inner membrane complex proteins (IMCps) that exhibit a conserved domain architecture comprising an N-terminal extracellular domain, a central transmembrane segment and a C-terminal cytoplasmic domain. Furthermore, all A-type genomes contain collagen-like proteins with glycine rich repeats (Gly–X–Y), forming triple helices potentially mediating adhesion to plant tissues [71].

In contrast, the P-type strains GOE and PENLEP lack collagen-like domains and instead encode VMPA-like proteins with multiple tandem spiralin-like domains. Spiralins, described as membrane lectins in *Spiroplasma* spp. [72], can mediate host interactions and as shown for Spiralin B, can couple host proliferation and vertical transmission, promoting long-term host–symbiont stability [73]. While only GOE encodes a spectrin-like protein that may stabilize membranes or mediate adhesion [74], and PENLEP encodes a structural maintenance of chromosome (Smc)-like protein, both were identified by OrthoFinder as orthologs of the collagen-like proteins. SRP1-like proteins, identified in POT, c1 and c4, encodes a phytoplasma effector recently shown to suppress the insect melanisation immune response, thereby facilitating persistent pathogen transmission [75]. These findings suggest a dual role for SRP1-like proteins as membrane-associated factors and active virulence determinants that remodel host physiology to promote vector-mediated spread [75,76].

Besides the IDPs and lineage-specific proteins, all the genomes encode hemolysin-like proteins, including HlyC-like, and TlyC-like variants. HlyC harbours hemolysin III-related domain and is likely involved in phytoplasma virulence [4]. In contrast, TlyC carries additional CorC-HlyC domains, functions as an NTPase, and binds the plant protein Toc33, a component of the chloroplast protein import machinery, suggesting a possible role in host–organelle interaction and nucleotide acquisition [77]. Also present are P38-like adhesin-like membrane proteins, first described in ‘*Ca*. P. solani’, whose interaction with hosts depends on the conserved Mollicutes adhesin motif (MAM) [78], and superoxide dismutase (SOD), which provides protection against reactive oxygen species (ROS) generated during host immune responses and within insect vectors [79].

#### 3.5.3. Secretion of Effectors

A focused analysis of the secreted aster yellows witches’-broom (AYWB) proteins (SAPs) [4] in the analysed A- and P-type phytoplasmas revealed a highly specialized repertoire, restricted to SAP05, SAP11, SAP21, and SAP54 (Figure 9). In the A-type strain POT, a total of 27 putative secreted proteins were identified (Supplementary Table S5), three of which could be assigned to SAPs. Within this set, all effectors except SAP05 were present, whereas c1 and c4 maintain the complete set of four SAPs. Strain o3 encodes only SAP54, and c5 contains SAP21 and SAP54. In the P-type strains GOE and PENLEP, SAP05, SAP11, and SAP54 are present, but SAP21 was not identified.

The functions of these SAPs have been studied in recent years, revealing distinct but also complementary modes of host manipulation. SAP05 [80] promotes host proliferation and induces abnormal leaf morphogenesis, whereas SAP11 [81] destabilizes class II teosinte Branched1/Cycloidea/Proliferating cell factor (TCP) transcription factors (TFs), driving proliferation and witches’ broom formation. SAP21, detected in certain ‘*Ca.* P. aurantifolia’ strains and co-expressed with SAP11 [82], remains functionally uncharacterized but occurs selectively in specific A-type lineages, suggesting a role in host-specific adaptation. SAP54 [83] targets floral MADS-domain proteins for degradation, triggering phyllody symptoms that prolong vegetative growth and enhance vector acquisition efficiency.

**Figure 10:**
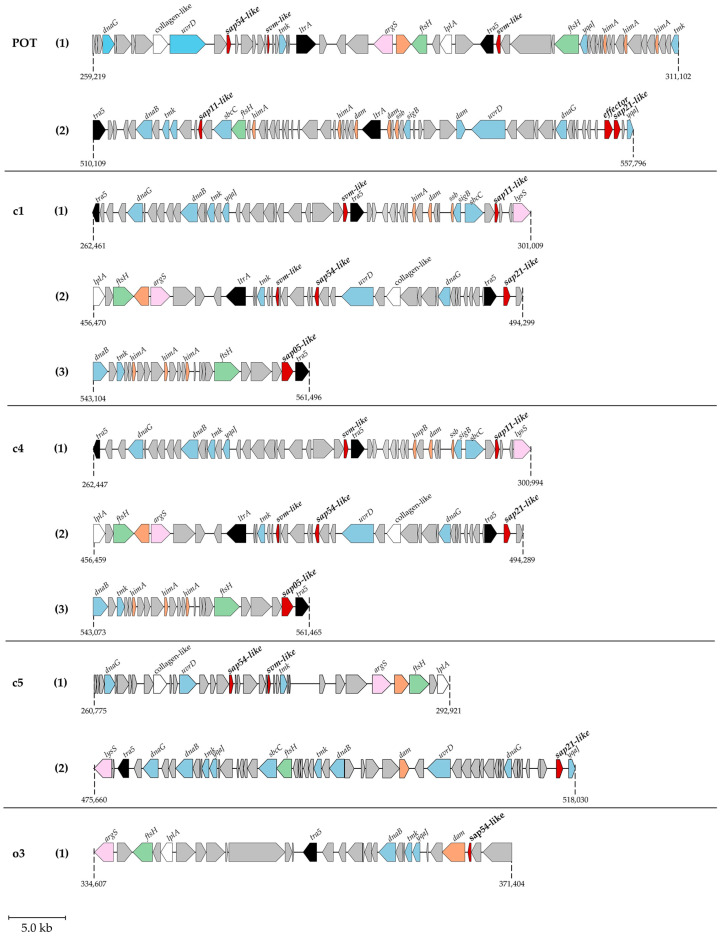
Genomic context of the effectors for five complete 16SrXII-A genomes. Effector proteins (red), DNA regulation and packaging proteins (orange), replication and recombination proteins (blue), hypothetical proteins (grey), translation proteins (purple), mobile genetic elements (black), and metabolic or structural accessory factors (white) are shown with their orientation in the chromosome. Gene names are indicated above the corresponding loci. Numbering indicates individual PMUs within the genomes.

Like GOE, PENLEP encodes a complete phytoplasma pathogenicity island (PPAI) carrying multiple effectors [20], whereas POT lacks such a contiguous transposon but displays an effector organization resembling other A-type genomes (Figure 10), with effectors located at separate chromosomal sites yet flanked by transposon-associated genes. In POT, the effector genes SAP11, SAP21, and SAP54 are located within two separate genomic segments: PMU1 (259–311 kb), representing 6.25% of the chromosome, and PMU2 (510–557 kb), representing 5.65%. We depict the effector-associated regions as follows: a regulation and DNA packaging module (*ssb*, *dam*, *himA*), and a replication and recombination module (*yqaJ*, *tmk*, *dnaB*, *dnaG*, *uvrD*, *sbcC*, *sigB*). Additional functional categories include a protein quality control module (*ftsH*), a translation module (*argS* and *lysS*), an effector module (effector proteins) and accessory module (collagen-like and *lpla*). Mobile genetic elements (*tra5*, *ltrA*) together with numerous hypothetical proteins are interspersed within these regions. While c1 and c4 each harbour three effector-associated regions with an almost identical organization, c5 and POT contain only two, and o3 a single one. In all genomes except o3, the SAP54-like protein is directly flanked by hypothetical proteins, followed by *uvrD*, *tmk*, and an *svm*-like gene; moreover, collagen-like proteins are located in close vicinity. To note, o3 differs from this canonical arrangement, as both the number and order of effector-associated regions are reduced, and several flanking modules are absent or rearranged. Among the examined genomes, POT is most similar to c5 in overall effector gene organization.

## 4. Discussion

The complete genome sequence of the ‘*Ca.* P. solani’ strain POT presented here adds a further representative to the number of complete stolbur phytoplasma genomes available to date and provides novel insights into intra-lineage diversification and virulence repertoires, representing the first complete A-type genome transmitted by *H. obsoletus* from potato fields in Germany.

Given that the placement of POT within the broader phylogenetic context requires a robust species assignment, it is essential to consider how taxonomic boundaries in phytoplasmas are currently defined. The delineation of species boundaries in phytoplasmas has long relied on *16S rRNA* identity and RFLP analysis [9]. While robust for initial classification, these markers can complicate species assignment, as newly identified strains may exhibit identical *16S rRNA* sequences despite belonging to different taxa [84]. To overcome these limitations, recent taxonomic frameworks incorporate genome-wide ANI and full-length *16S rRNA* gene comparison, applying thresholds of 95% ANI and 98.65% *16S rRNA* identity [10]. In our analysis, ANI confirmed the close relatedness of A-type strains, while clearly separating them from representatives of the P-type lineage. Earlier work [20], based on a single P-type genome, already demonstrated this divergence through genome-wide ANI and phylogenetic inference from conserved markers such as *tuf* and partial *16S rRNA*. Our *16S rRNA* analysis incorporated trimmed reference taxa beyond the 16SrXII subgroup, further demonstrating that 16SrXII-P forms a distinct lineage clearly separated from other taxa [17]. Building on this, we embedded the separation within a comprehensive phylogenomic framework. Using all currently available complete phytoplasma genomes, we reconstructed a phylogeny from 41 universally conserved SCOs, placing POT unambiguously within the 16SrXII-A cluster. From this comprehensive dataset, we observed a cBUSCO separation around 97.8%, which was applied as a complementary genomic metric for species delineation within phytoplasmas. This genome-wide approach was not only able to distinguish the P-Type from the A-type, but more importantly, it resolved the nettle-originated strain o3 as a separate lineage from the bindweed-derived strains c1, c4, and c5 as well as from POT. The distinct position of o3 is further reflected in its slightly lower ANI values and the divergent genome organization, whereas POT shares a closer genomic architecture with the bindweed-associated A-type strains, underlining their tighter evolutionary relationship. The high congruence between cBUSCO, ANI, and phylogenomic topology underscores the value of integrating multiple genome-wide approaches in phytoplasma taxonomy, particularly for lineages where *16S rRNA* gene identity alone fails to capture evolutionary divergence [10,84].

Beyond its phylogenetic placement, the genome of POT reflects the characteristic architecture of 16SrXII-A, combining a conserved metabolic core with a comparatively reduced mobilome []. Its metabolism comprises an almost complete glycolytic pathway coupled to ATP-generating acetogenesis, alongside glycerophospholipid synthesis for membrane maintenance, consistent with other phytoplasma lineages [20,64]. Within this framework, o3 is of particular interest as it is able to encode GtfA. Sucrose phosphorolysis by GtfA yields glucose-1-phosphate and fructose, providing a direct entry into carbon metabolism [85,86]. Without Pgm, channelling these products into glycolysis could rely on alternative pathways such as activity of unspecific haloacid dehalogenase (HAD) superfamily subfamily IIb-containing enzymes. These enzymes are predicted to act as phosphatases or phosphotransferases [87] on substrates common in phytoplasma environments, potentially enabling the use of trehalose-6-phosphate [6] and the conversion of fructose to fructose-6-phosphate for entry into glycolysis [69]. While *gtfA* is retained as a functional gene only in o3 but occurs as pseudogene in other stolbur genomes, indicating host-specific relevance that remains to be clarified.

Alongside metabolic features, stolbur genomes are defined by their effector repertoires. In POT, effectors are dispersed across the chromosome and frequently positioned adjacent to transposon-associated genes, yet without forming the *tra5*-flanked transposon-embedded pathogenicity islands described for P-type genomes such as GOE [20]. The PMUs in POT retain the core genes first defined in ‘*Ca*. P. asteris’ AY-WB [4], with only minor local rearrangements, suggesting evolutionary conservation of these mobile elements within 16SrXII-A lineages. Both effector-associated regions are similar in size but differ in gene content. The first one harbours SAP54-like and two potential SVM-like proteins, whereas second one contains SAP11, SAP21, and an effector pseudogene. Notably, only the last one carries both *sbcC* and *dnaB*, two of the core PMU genes implicated in replication and mobilization [4,88], suggesting that this PMU may retain a higher potential for genomic rearrangement. This organization, together with the reduced number of phage-derived regions, points towards lower genome plasticity and potentially greater structural stability in A-type lineages. Across stolbur genomes, the set of effectors varies as GOE, PENLEP, c1, and c4 encode SAP11, SAP54, and SAP05, whereas c5 carries SAP21 and SAP54 and o3 only SAP54 [20]. In line with this, SAP54 is the only effector consistently conserved across all stolbur genomes examined, underlining its potential central role in stolbur pathogenesis. SAP54 targets MADS-box transcription factors and induces the formation of leaf-like flowers in *Arabidopsis thaliana*, thereby disrupting reproduction of the host plant [83]. The virescence symptoms observed in *C. roseus* infected with POT match with this mode of action. While SAP54 has further been suggested to influence insect vector behaviour and fertility [83], such effects have not been demonstrated for the key stolbur vectors *H. obsoletus* and *P*. *leporinus* [20]. Further studies are required to clarify its ecological relevance in vector–pathogen interactions.

Apart from effector variability, diversity is also reflected in a broader repertoire of membrane-associated and conserved putative virulence factors that could contribute to host colonization and vector transmission. All examined stolbur genomes encode a conserved set of IMPs, together with VMP1-like and STAMP homologs, underscoring their central role in mediating host–vector interactions and evading host immunity [20,89]. As orthologs of the collagen-like proteins, GOE and PENLEP further encode spiralinB-like, spectrin-like and smc-like proteins, that may contribute to membrane stabilization or host–cell adhesion [73,74]. Notably, POT encodes collagen-like proteins with domains annotated as quinoprotein-like alcohol dehydrogenase based on structural similarity, suggesting lineage-specific diversification of membrane-associated proteins. However, functional implications remain to be experimentally validated. Quinoprotein alcohol dehydrogenases are typically pyrroloquinoline quinone (PQQ)-dependent enzymes catalysing their periplasmic oxidation in periplasm of Gram-negative bacteria and could contribute to nutrient acquisition and detoxification [90,91]. SRP1, a member of the SRP protein family, was restricted to POT, c1, and c4, and given the role of SRP-like proteins in insect immunity [75], including melanisation cascades, this distribution may indicate lineage specific adaptation to vector defence systems.

Beyond these, all strains encode a conserved set of putative virulence factors, including the apoptosis regulator Bax inhibitor-1, SOD, the adhesin-like protein P38, and the hemolysin family proteins HlyC and TlyC. The ubiquitous presence of Bax inhibitor-1 across all genomes suggests a conserved function in suppressing programmed cell death in host tissues [92,93,94], potentially extending the period for available transmission. Under stress conditions of the host, such as those triggered by pathogenic bacterial infection, huge amounts of ROS are generated [95,96]. SOD is likely to act as a primary line of defence during plant immune responses and within insect vectors, a role known to be essential for the persistence of other mollicutes in oxidative environments [79,97]. P38, identified as an adhesin-like protein, may facilitate attachment to vector or plant cell surfaces [78], thereby potentially influencing transmission efficiency. The hemolysin-like proteins HlyC and TlyC, although less well characterized in phytoplasmas, are intriguing candidates for mediating membrane interactions or nutrient acquisition from host cells [77].

The genome of strain POT reveals lineage-specific features in mobile elements, effector repertoires, and membrane-associated virulence factors that are consistent with lineage-specific adaptation and may be involved in host interaction processes linked to persistence, symptom development, and transmission. Because phytoplasmas are phloem-limited and obligately insect-transmitted, stolbur spread is driven by the ecology of its insect vectors and their host associations. *H. obsoletus* populations drive transmission cycles through weed reservoirs such as bindweed and nettle, from which the pathogen is transferred to crops [26]. Field studies link the occurrence of *H. obsoletus* to environmental factors such as soil properties and precipitation [98], and demonstrate plant specialization rather than broad polyphagy [26], with reduced vector pressure achieved by targeted nettle removal in affected agroecosystems [99]. In contrast, *P. leporinus* persists in sugar beet– or potato–wheat rotations, developing on beet and potato, with nymphs overwintering in the soil during the wheat phase until suitable hosts are replanted [100]. Transmission is stage-dependent, since adults fail to acquire phytoplasmas from foliage, whereas nymphs feeding on infected roots efficiently develop into transmitting adults, with competence increasing from the fourth to fifth instar [101]. Such ecological specialization could explain the spread of stolbur vectors in Central Europe, and future warming is likely to increase their abundance and advance emergence, underscoring the need to integrate genomic and ecological perspectives to resolve adaptation and epidemiology in agroecosystems.

## 5. Conclusions

This study presents the complete genome of the 16SrXII-A strain POT and its comparative analysis with all currently available complete stolbur phytoplasmas. By applying a cBUSCO threshold across all currently available complete phytoplasma genomes, we introduce a complementary genomic metric for species delineation that is stable across phytoplasma lineages and aligns with established genome-wide ANI. Both approaches consistently resolve the taxonomic boundaries between the A- and P-type lineages. Effectors and prominent membrane proteins emerge as central drivers of phytoplasma adaptation, shaping interactions with plant hosts and insect vectors. Despite a conserved metabolic and immunodominant protein repertoire, more lineage-specific differences in mobile genetic elements, membrane-associated proteins, and virulence repertoires point to functional diversification. Such genomic variation likely underpins differences in host range, symptom expression, and transmission efficiency, providing a robust framework for future studies on stolbur phytoplasma adaptation and epidemiology.

## Figures and Tables

**Table 1:** Comparison of genomic features between strain POT and other complete 16SrXII phytoplasma genomes.

Subgroup	16SrXII-A					16SrXII-P	
Strain	POT	c1	c4	c5	o3	GOE	PENLEP
Chromosome size [bp]	832,614	751,320	751,188	824,084	973,640	704,525	700,012
G + C content [%]	28.21	28.37	28.37	28.07	28.58	26.17	26.2
CDS coding	740	719	724	807	1000	663	637
Coding density [CDS/kb]	0.888	0.956	0.963	0.979	1.027	0.941	0.908
CDS coding length [kb]	0.860	0.810	0.804	0.786	0.723	0.808	0.864
Hypothetical proteins	236	350	356	271	200	118	280
rRNA Operons	2	2	2	2	2	2	2
tRNAs	32	32	32	32	32	32	32
ncRNAs	2	2	2	2	2	2	2
tmRNAs	1	1	1	1	1	1	1
Host for reconstruction	*Catharanthus roseus*	*Bindweed*	*Bindweed*	*Bindweed*	*Stinging nettle*	*Pentastiridius leporinus*	*Pentastiridius leporinus*
Data source	CM135798.1	CP103788	CP103787	CP103786	CP103785	CP155828	[54]

**Table 2:** Pairwise cBUSCO identities (in %) between seven complete stolbur phytoplasma genomes. Values exceeding the species threshold of 97.8% are shown in bold.

Subgroup		16SrXII-A					16SrXII-P		16SrXII-B/C	
	Strain	c1	c4	c5	POT	o3	GOE	PENLEP	PAa	NZSb11
**16SrXII-A**	c1	**-**	**100.0**	**99.96**	**99.99**	**99.79**	84.81	84.80	82.32	82.38
	c4	**100.0**	**-**	**99.96**	**99.99**	**99.79**	84.81	84.80	82.32	82.38
	c5	**99.96**	**99.96**	**-**	**99.96**	**99.76**	84.80	84.79	82.32	82.38
	**POT**	**99.99**	**99.99**	**99.96**	**-**	**99.80**	84.81	84.80	82.33	82.39
	o3	**99.79**	**99.79**	**99.76**	**99.80**	**-**	84.79	84.78	82.29	82.36
**16SrXII-P**	GOE	84.81	84.81	84.80	84.81	84.79	-	**99.991**	85.54	85.56
	PENLEP	84.80	84.80	84.79	84.80	84.78	**99.991**	-	85.53	85.55
**16SrXII-B/C**	PAa	82.32	82.32	82.320	82.33	82.29	85.54	85.53	-	**99.73**
	NZSb11	82.38	82.38	82.38	82.39	82.36	85.56	85.55	**99.73**	-

Grey shading highlights subgroup assignment based on cBUSCO identities exceeding the threshold.

**Table 3:** Pairwise shared orthogroups between seven complete stolbur phytoplasma genomes.

Subgroup		16SrXII-A					16SrXII-P	
	Strain	c1	c4	c5	POT	o3	GOE	PENLEP
**16SrXII-A**	c1	**-**	626	594	534	556	431	434
	c4	626	**-**	593	537	555	432	434
	c5	594	593	**-**	541	562	430	434
	**POT**	534	537	541	**-**	523	431	434
	o3	556	555	562	523	**-**	426	431
**16SrXII-P**	GOE	431	432	430	431	426	**-**	502
	PENLEP	434	434	434	434	431	502	**-**

**Table 4:** Impact of multi-copy genes in complete stolbur phytoplasma genomes.

Subgroup	Strain	Number of Multi-Copy Genes	% of Total Genes	Total Length (bp)	Chromosome Fraction (%)
**16SrXII-A**	c1	135	18.78	92,445	12.30
	c4	139	19.20	87,462	11.64
	c5	214	26.52	141,039	17.11
	**POT**	235	31.76	172,948	20.77
	o3	413	41.30	293,316	30.13
**16SrXII-P**	GOE	157	23.68	93,876	13.32
	PENLEP	154	24.18	93,282	13.33

## Data Availability

The sequence and annotation of ‘*Ca.* P. solani’ POT were deposited in the GenBank database under accession number CM135798.1.

## Supplementary Materials

The following supporting information can be downloaded at: https://www.mdpi.com/article/10.3390/microorganisms14010226/s1, Table S1: List of complete phytoplasma genomes; Table S2: BUSCO-based set of 41 universally conserved SCOs used in phylogenomic reconstruction; Table S3: cBUSCO identity matrix (in %) across complete phytoplasma genomes; Table S4: BLASTP matrix (in %) across complete stolbur phytoplasma genomes based on STAMP; Table S5: Predicted POT-secreted proteins (POTSs).
