# Supplementary material for: Comparative Genome Analysis of 16SrXII-A ‘Candidatus Phytoplasma solani’ POT Transmitted by Hyalesthes obsoletus
Source: Microorganisms. 2026 Jan 19;14(1):226. doi: 10.3390/microorganisms14010226 (PMC12843639; doi:10.3390/microorganisms14010226)
Supplement: Supplementary file 1 [file microorganisms-14-00226-s001.zip › microorganisms-4078608-supplementary.pdf]

Table S1. List of complete phytoplasma genomes.

Species	Strain	Genome size (kb)	Accession number
'Ca. P. asteris'	AYWB	706.57	CP000061
'Ca. P. asteris'	M33	657.32	CP128397
Onion yellows phytoplasma	OY-M	853.09	AP006628
'Ca. P. asteris'	De Villa	600.12	CP035949
Maize bushy stunt phytoplasma	M3	576.12	CP015149
Paulownia witches'-broom phytoplasma	Zhengzhou	891.64	CP066882
'Ca. P. asteris'	QS2022	834.30	CP120448
Mulberry dwarf phytoplasma	MDGZ-01	622.36	CP085837
'Ca. P. asteris'	M8	772.69	CP128414
Rapeseed phyllody phytoplasma	RP166	829.55	CP055264
'Ca. P. solani'	o3	973.64	CP103785
'Ca. P. solani'	c5	824.08	CP103786
'Ca. P. solani'	POT	832.61	This study
'Ca. P. solani'	c1	751.32	CP103788
'Ca. P. solani'	c4	751.19	CP103787
'Ca. P. solani'	GOE	704.52	CP155828
'Ca. P. solani'	PENLEP	700.012	[54]
'Ca. P. australiense'	PAa	879.96	AM422018
Strawberry lethal yellows phytoplasma	NZSb11	959.78	CP002548
'Ca. P. asiaticum'	PR34	614.57	CP097206
'Ca. P. australasiaticum'	PT202407	399.46	CP171835
'Ca. P. australasiaticum'	SPWB	602.82	CP171825
'Ca. P. australasiaticum subsp. australasiaticum'	PR08	588.75	CP060385
'Ca. P. australasiaticum'	NCHU2022	632.99	CP097312
'Ca. P. australasiaticum subsp. taiwanense'	NCHU2014	635.58	CP040925
'Ca. P. australasiaticum'	WF_GM2021	633.00	CP133702
'Ca. P. citri'	TB2022	670.07	CP120449
'Ca. P. mali'	AT	601.94	CU469464
'Ca. P. rubi'	RS	762.25	CP114006
'Ca. P. vitis'	CH	654.22	CP097583
'Ca. P. ziziphi'	Jwb-nky	750.80	CP025121
'Prunus avium' virescence phytoplasma	SCV-TA2020	775.34	CP069156
'Ca. P. ziziphi'	Hebei-2018	764.11	CP091835
'Ca. P. luffae'	NCHU2019	769.14	CP054393
'Ca. P. fraxini'	AshY1	598.52	CP146843
Phytoplasma sp.	ArAWB-2021	554.36	CP131022
'Ca. P. pruni'	PR2021	705.14	CP119306
'Ca. P. oryzae'	HN2022	511.27	CP116038
'Ca. P. cynodontis'	GY2015	498.92	CP126225
'Ca. P. sacchari'	SCWL1	538.95	CP115156

Table S2. BUSCO-based set of 41 universally conserved SCOs used in phylogenomic reconstruction.

Nr.	OrthoDB-ID	Description
1	10003at31969	Ribosomal protein L35
2	1459at31969	Translation initiation factor IF-2
3	1640at31969	Glycine--tRNA ligase
4	1702at31969	Elongation factor Tu
5	2086at31969	GTPase Der
6	2445at31969	Serine--tRNA ligase
7	2999at31969	Ribosomal protein L2
8	320at31969	Elongation factor G
9	3395at31969	Cysteine--tRNA ligase
10	3928at31969	tRNA-specific 2-thiouridylase
11	4364at31969	Chaperone protein DnaJ
12	437at31969	Lon protease
13	4488at31969	Ribosomal protein L1
14	4792at31969	K Homology domain
15	510at31969	Elongation factor 4
16	5532at31969	Methionine aminopeptidase
17	5908at31969	Ribosomal protein L5
18	6279at31969	Ribosomal protein L13
19	6347at31969	Uridylate kinase
20	6579at31969	Ribosomal protein L11, C-terminal
21	6611at31969	Inorganic pyrophosphatase
22	6835at31969	Ribosomal protein L16
23	7077at31969	Ribosomal protein S5
24	7534at31969	Ribosomal protein S8
25	7632at31969	Ribosomal protein S7 domain
26	7636at31969	Ribosome recycling factor
27	7986at31969	Ribosomal protein L15
28	8036at31969	Ribosomal protein S9
29	8165at31969	Ribosomal protein S11
30	8205at31969	Ribosomal protein L14P
31	832at31969	Leucine--tRNA ligase
32	8340at31969	Ribosomal protein L27
33	8630at31969	Ribosomal protein L17
34	8685at31969	Thymidylate kinase
35	885at31969	Chaperone protein DnaK
36	8971at31969	Ribosomal protein S19, bacterial-type
37	9413at31969	Ribosomal protein S17/S11
38	9452at31969	Ribosomal protein L21
39	9604at31969	Ribosomal protein L18
40	9756at31969	Ribosomal protein L31
41	9763at31969	Ribosomal protein L28/L24

Table S3. cBUSCO identity matrix (in %) across complete phytoplasma genomes.

	AYWB	M33	OY-M	De Villa	M3	Zhengzhou	QS2022	MDGZ-01	M8	RP166	o3	c5	POT (This study)	c1	c4	GOE	PENLEP	Paa	NZSb11	PR34	PT202407	spwb	PR08	NCHU2022	NCHU2014	WF_GM2021	TB2022	AT	RS	CH	Jwb-nky	SCV-TA2020	Hebei-2018	NCHU2019	AshY1	ArAWB-2021	PR2021	HN2022	GY2015	SCWL1
'Ca. P. asteris' AYWB (CP000061)	100.0	99.806	95.371	95.647	95.507	95.664	95.981	95.981	95.955	95.963	79.329	79.365	79.365	79.365	79.365	79.223	79.214	78.26	78.287	56.505	60.959	58.797	56.548	56.53	56.556	56.548	56.548	64.929	61.336	61.754	61.02	61.246	61.597	60.994	60.994	61.366	64.472	58.688	58.877	59.13
'Ca. P. asteris' M33 (CP128397)	99.806	100.0	95.341	95.647	95.507	95.655	95.955	95.963	95.937	95.946	79.285	79.321	79.321	79.321	79.321	79.214	79.205	78.26	78.287	56.439	60.959	58.872	56.487	56.469	56.496	56.487	56.487	64.885	61.301	61.719	60.985	61.209	61.574	60.968	60.994	61.378	64.463	58.697	58.821	59.159
Onion yellows phytoplasma OY-M (AP006628)	95.371	95.341	100.0	98.746	98.589	98.477	99.037	99.037	99.485	99.485	78.465	78.514	78.514	78.514	78.514	78.01	78.002	79.329	79.374	55.849	58.496	58.563	55.915	55.889	55.915	55.906	55.906	65.685	62.944	63.3	62.087	62.982	63.079	62.136	60.122	62.32	66.041	57.895	60.542	58.308
'Ca. P. asteris' De Villa (CP035949)	95.647	95.647	98.746	100.0	99.261	98.267	98.416	98.61	98.566	98.575	79.413	79.448	79.448	79.448	79.448	79.478	79.47	78.408	78.435	56.45	58.654	58.72	56.525	56.499	56.525	56.516	56.516	64.818	61.2	61.632	60.856	61.088	61.453	60.903	60.842	61.205	64.33	58.7	58.988	59.017
Maize bushy stunt phytoplasma M3 (CP015149)	95.507	95.507	98.589	99.261	100.0	98.003	98.1	98.364	98.302	98.311	79.273	79.308	79.308	79.308	79.308	79.354	79.345	78.32	78.346	56.403	58.433	58.63	56.459	56.433	56.459	56.45	56.45	64.745	61.072	61.504	60.734	60.94	61.331	60.864	60.781	61.162	64.289	58.622	58.988	59.069
Paulownia witches'-broom phytoplasma Zhengzhou (CP066882)	95.664	95.655	98.477	98.267	98.003	100.0	98.422	98.695	98.748	98.757	79.589	79.625	79.625	79.625	79.625	79.5	79.491	78.576	78.602	56.472	58.23	58.633	56.486	56.459	56.486	56.477	56.477	64.882	61.017	61.48	60.685	60.935	61.292	60.853	60.773	61.171	64.337	58.654	59.076	59.03
'Ca. P. asteris' QS2022 (CP120448)	95.981	95.955	99.037	98.416	98.1	98.422	100.0	98.827	98.801	98.81	79.692	79.727	79.727	79.727	79.727	79.504	79.495	78.642	78.669	56.581	58.407	58.767	56.53	56.504	56.52	56.511	56.511	64.91	61.198	61.651	60.929	61.138	61.518	60.936	60.912	61.293	64.508	58.697	59.043	59.095
Mulberry dwarf phytoplasma MDGZ-01 (CP085837)	95.981	95.963	99.037	98.61	98.364	98.695	98.827	100.0	99.083	99.092	79.56	79.595	79.595	79.595	79.595	79.504	79.495	78.669	78.695	56.548	58.363	58.791	56.544	56.518	56.544	56.535	56.535	64.872	61.189	61.634	60.866	61.099	61.473	60.939	60.857	61.236	64.393	58.633	59.043	59.069
'Ca. P. asteris' M8 (CP128414)	95.955	95.937	99.485	98.566	98.302	98.748	98.801	99.083	100.0	99.991	79.736	79.771	79.771	79.771	79.771	79.578	79.569	78.687	78.713	56.592	58.584	58.866	56.596	56.57	56.596	56.587	56.587	64.98	61.187	61.649	60.841	61.101	61.457	60.95	60.868	61.307	64.488	58.723	59.067	58.998
Rapeseed phyllody phytoplasma RP166 (CP055264)	95.963	95.946	99.485	98.575	98.311	98.757	98.81	99.092	99.991	100.0	79.745	79.78	79.78	79.78	79.78	79.587	79.578	78.696	78.722	56.601	58.584	58.866	56.605	56.579	56.605	56.596	56.596	64.989	61.189	61.658	60.85	61.111	61.465	60.959	60.877	61.316	64.496	58.731	59.076	59.006
'Ca. P. solani' o3 (CP103785)	79.338	79.294	78.451	79.461	79.32	79.582	79.739	79.616	79.773	79.782	100.0	99.76	99.796	99.787	99.787	84.749	84.74	82.197	82.259	56.333	65.909	58.818	56.535	56.509	56.535	56.526	56.526	65.305	61.055	61.374	60.704	60.654	61.181	60.669	60.819	60.898	63.622	58.58	58.799	58.657
'Ca. P. solani' c5 (CP103786)	79.373	79.329	78.5	79.496	79.355	79.618	79.775	79.651	79.808	79.817	99.76	100.0	99.964	99.956	99.956	84.758	84.749	82.223	82.285	56.351	65.909	58.879	56.553	56.526	56.553	56.544	56.544	65.323	61.046	61.365	60.704	60.645	61.172	60.66	60.766	60.845	63.587	58.589	58.799	58.709
'Ca. P. solani' POT (This study)	79.373	79.329	78.5	79.496	79.355	79.618	79.775	79.651	79.808	79.817	99.796	99.964	100.0	99.991	99.991	84.766	84.758	82.232	82.294	56.359	65.909	58.864	56.561	56.535	56.561	56.553	56.553	65.34	61.064	61.383	60.712	60.663	61.19	60.677	60.792	60.871	63.604	58.606	58.816	58.701
'Ca. P. solani' c1 (CP103788)	79.373	79.329	78.5	79.496	79.355	79.618	79.775	79.651	79.808	79.817	99.787	99.956	99.991	100.0	100.0	84.766	84.758	82.223	82.285	56.359	65.909	58.864	56.561	56.544	56.57	56.561	56.561	65.34	61.064	61.383	60.712	60.663	61.19	60.677	60.792	60.871	63.604	58.606	58.816	58.701
'Ca. P. solani' c4 (CP103787)	79.373	79.329	78.5	79.496	79.355	79.618	79.775	79.651	79.808	79.817	99.787	99.956	99.991	100.0	100.0	84.766	84.758	82.223	82.285	56.359	65.909	58.864	56.561	56.544	56.57	56.561	56.561	65.34	61.064	61.383	60.712	60.663	61.19	60.677	60.792	60.871	63.604	58.606	58.816	58.701
'Ca. P. solani' GOE (CP155828)	79.24	79.232	79.442	79.522	79.398	79.518	79.539	79.539	79.613	79.622	84.793	84.802	84.811	84.811	84.811	100.0	99.991	85.493	85.52	56.219	60.0	58.374	56.266	56.24	56.248	56.24	56.24	64.922	60.833	61.15	60.644	60.66	61.085	60.164	60.476	60.442	63.099	58.483	58.725	58.512
'Ca. P. solani' PENLEP (Vogel, 2025)	79.232	79.223	79.428	79.514	79.389	79.509	79.53	79.53	79.604	79.613	84.784	84.793	84.802	84.802	84.802	99.991	100.0	85.485	85.511	56.219	60.0	58.374	56.266	56.24	56.248	56.24	56.24	64.913	60.825	61.141	60.635	60.65	61.076	60.156	60.484	60.433	63.09	58.483	58.725	58.504
'Ca. P. australiense' PAa (AM422018)	78.351	78.351	78.793	78.498	78.409	78.63	78.732	78.758	78.776	78.785	82.294	82.32	82.329	82.32	82.32	85.538	85.529	100.0	99.734	55.901	58.61	58.182	56.096	56.105	56.122	56.114	56.114	64.558	60.619	61.034	60.314	60.577	60.825	60.442	60.351	60.59	63.446	58.057	58.367	58.381
Strawberry lethal yellows phytoplasma NZSb11 (CP002548)	78.377	78.377	78.837	78.524	78.436	78.657	78.758	78.785	78.803	78.812	82.356	82.382	82.391	82.382	82.382	85.564	85.555	99.734	100.0	55.853	58.566	58.106	56.035	56.035	56.052	56.043	56.043	64.514	60.584	60.999	60.279	60.549	60.789	60.416	60.333	60.572	63.411	58.066	58.358	58.354
'Ca. P. asiaticum' PR34 (CP097206)	56.415	56.376	55.816	56.364	56.316	56.332	56.477	56.462	56.558	56.567	56.355	56.356	56.365	56.365	56.365	56.243	56.243	55.804	55.733	100.0	90.958	90.974	90.054	89.992	90.001	90.01	90.01	56.966	60.436	60.717	60.221	60.65	60.766	59.627	60.186	60.184	62.301	59.727	59.986	59.768
'Ca. P. australasiaticum' PT202407 (CP171835)	60.959	60.959	58.496	58.654	58.433	58.23	58.407	58.815	58.584	58.584	65.909	65.909	65.909	65.909	65.909	60.0	60.0	58.61	58.566	90.958	100.0	98.974	98.885	98.974	98.93	98.974	98.974	58.563	64.028	64.295	64.146	63.09	64.146	63.644	63.97	64.286	66.667	65.426	65.018	64.457
'Ca. P. australasiaticum' SPWB (CP171825)	58.697	58.742	58.486	58.552	58.462	58.39	58.583	58.622	58.788	58.788	58.831	58.891	58.876	58.876	58.876	58.398	58.398	58.065	57.99	90.974	98.974	100.0	99.672	100.0	100.0	100.0	100.0	58.341	62.545	62.759	61.746	62.479	62.566	61.387	61.8	62.207	64.272	62.822	62.223	62.074
'Ca. P. australasiaticum' PR08 (CP060385)	56.435	56.417	55.881	56.448	56.399	56.355	56.444	56.466	56.571	56.579	56.567	56.585	56.594	56.594	56.594	56.303	56.303	56.028	55.967	90.054	98.885	99.672	100.0	99.761	99.77	99.779	99.779	56.651	60.672	60.945	60.344	61.261	60.845	59.851	60.361	60.392	62.525	60.007	60.178	60.063
'Ca. P. australasiaticum' NCHU2022 (CP097312)	56.417	56.4	55.855	56.421	56.356	56.329	56.417	56.44	56.545	56.553	56.532	56.558	56.567	56.567	56.567	56.277	56.277	56.037	55.975	89.992	98.974	100.0	99.761	100.0	99.973	99.982	99.982	56.669	60.637	60.919	60.29	61.261	60.773	59.798	60.296	60.319	62.499	59.955	60.125	60.054
'Ca. P. australasiaticum' NCHU2014 (CP040925)	56.444	56.426	55.881	56.448	56.382	56.355	56.444	56.466	56.571	56.579	56.558	56.585	56.594	56.594	56.594	56.286	56.286	56.054	55.993	90.001	98.93	100.0	99.77	99.973	100.0	99.991	99.991	56.674	60.655	60.936	60.307	61.261	60.791	59.816	60.317	60.336	62.516	59.972	60.143	60.072
'Ca. P. australasiaticum' WF_GM2021 (CP133702)	56.435	56.417	55.873	56.439	56.39	56.347	56.435	56.457	56.562	56.571	56.55	56.576	56.585	56.585	56.585	56.277	56.277	56.046	55.984	90.01	98.974	100.0	99.779	99.982	99.991	100.0	100.0	56.674	60.646	60.928	60.298	61.261	60.782	59.807	60.308	60.327	62.508	59.963	60.134	60.063
'Ca. P. citri' TB2022 (CP120449)	56.435	56.417	55.873	56.439	56.39	56.347	56.435	56.457	56.562	56.571	56.55	56.576	56.585	56.585	56.585	56.277	56.277	56.046	55.984	90.01	98.974	100.0	99.779	99.982	99.991	100.0	100.0	56.674	60.646	60.928	60.298	61.261	60.782	59.807	60.308	60.327	62.508	59.963	60.134	60.063
'Ca. P. mali' AT (CU469464)	64.923	64.879	64.104	64.867	64.822	64.877	64.922	64.902	64.993	65.001	65.209	65.226	65.244	65.244	65.244	64.923	64.914	64.566	64.522	57.08	56.565	58.419	56.697	56.715	56.724	56.724	56.724	100.0	62.183	62.347	61.794	61.999	62.218	61.324	61.564	61.552	63.552	59.916	60.362	60.201
'Ca. P. rubi' RS (CP114006)	61.343	61.317	62.989	61.234	61.099	61.009	61.231	61.223	61.214	61.223	61.078	61.07	61.087	61.087	61.087	60.833	60.818	60.625	60.59	60.444	64.028	62.494	60.651	60.616	60.634	60.625	60.625	62.104	100.0	97.69	92.741	94.249	94.001	74.172	79.658	80.032	69.55	66.162	66.879	66.129
'Ca. P. vitis' CH (CP097583)	61.77	61.744	63.329	61.683	61.555	61.47	61.668	61.659	61.674	61.683	61.397	61.388	61.406	61.406	61.406	61.15	61.141	61.0	60.965	60.743	64.295	62.79	60.954	60.928	60.942	60.936	60.936	62.317	97.69	100.0	93.711	94.991	94.99	74.468	80.282	80.73	70.09	66.507	67.103	66.498
'Ca. P. ziziphi' Jwb-nky (CP025121)	61.029	61.002	62.131	60.89	60.779	60.684	60.971	60.9	60.875	60.884	60.736	60.736	60.745	60.745	60.745	60.642	60.633	60.297	60.262	60.23	64.146	61.761	60.353	60.298	60.316	60.307	60.307	61.783	92.741	93.658	100.0	97.86	98.584	73.939	79.549	79.947	69.437	65.913	66.42	65.813
'Prunus avium' virescence phytoplasma SCV-TA2020 (CP069156)	61.255	61.227	63.014	61.115	60.966	60.924	61.174	61.126	61.128	61.137	60.708	60.708	60.708	60.708	60.708	60.639	60.63	60.573	60.545	60.65	63.09	62.496	61.261	61.261	61.261	61.261	61.261	61.958	94.249	94.991	97.86	100.0	99.361	74.315	80.079	80.406	69.982	66.667	67.626	66.11
'Ca. P. ziziphi' Hebei-2018 (CP091835)	61.618	61.592	63.123	61.487	61.364	61.291	61.561	61.506	61.49	61.499	61.196	61.187	61.204	61.204	61.204	61.074	61.065	60.808	60.773	60.775	64.146	62.581	60.854	60.782	60.799	60.791	60.791	62.181	94.001	94.937	98.584	99.361	100.0	74.639	80.436	80.887	70.123	66.617	67.018	66.503
'Ca. P. luffae' NCHU2019 (CP054393)	60.992	60.978	62.217	60.898	60.856	60.792	60.91	60.924	60.959	60.968	60.786	60.778	60.795	60.795	60.795	60.268	60.259	60.455	60.429	59.765	63.972	61.544	60.014	59.961	59.979	59.97	59.97	61.462	74.338	74.654	74.096	74.511	74.824	100.0	73.388	74.001	68.962	65.602	65.812	65.942
'Ca. P. fraxini' AshY1 (CP146843)	60.941	60.95	60.054	60.807	60.746	60.711	60.865	60.805	60.826	60.835	60.803	60.75	60.776	60.776	60.776	60.437	60.445	60.313	60.295	60.172	63.938	61.782	60.365	60.318	60.335	60.326	60.326	61.516	79.619	80.226	79.546	80.056	80.397	73.265	100.0	87.583	68.961	65.423	65.958	65.335
Phytoplasma sp. ArAWB-2021 (CP131022)	61.338	61.329	62.35	61.164	61.12	61.094	61.252	61.194	61.266	61.274	60.886	60.834	60.86	60.86	60.86	59.573	59.573	60.512	60.494	60.206	64.286	62.156	60.389	60.336	60.354	60.345	60.345	61.47	80.021	80.72	79.938	80.452	80.877	73.816	87.587	100.0	69.214	65.966	66.293	65.89
'Ca. P. pruni' PR2021 (CP119306)	64.406	64.397	66.118	64.313	64.273	64.285	64.491	64.401	64.514	64.523	63.705	63.67	63.687	63.687	63.687	63.099	63.09	63.435	63.4	62.359	66.667	64.332	62.601	62.575	62.592	62.583	62.583	63.522	69.585	70.125	69.474	69.982	70.161	68.799	68.987	69.301	100.0	68.052	68.869	68.452
'Ca. P. oryzae' HN2022 (CP116038)	58.736	58.745	57.897	58.728	58.65	58.694	58.699	58.673	58.725	58.733	58.647	58.655	58.673	58.673	58.673	58.564	58.564	58.01	58.018	59.801	65.426	62.703	59.991	59.939	59.957	59.948	59.948	59.927	66.188	66.524	65.933	67.025	66.641	65.635	65.48	66.036	68.175	100.0	79.579	79.134
'Ca. P. cynodontis' GY2015 (CP126225)	59.019	58.955	60.882	59.121	59.13	59.193	59.176	59.176	59.253	59.262	58.989	59.007	59.024	59.024	59.024	58.848	58.848	58.408	58.425	60.136	65.388	62.551	60.31	60.258	60.276	60.267	60.267	60.479	67.068	67.283	66.62	66.728	67.22	65.853	66.063	66.432	69.018	79.682	100.0	80.23
'Ca. P. sacchari' SCWL1 (CP115156)	59.303	59.303	58.473	59.18	59.206	59.159	59.25	59.224	59.196	59.205	58.857	58.909	58.901	58.901	58.901	58.715	58.706	58.488	58.462	59.82	64.502	62.289	60.169	60.152	60.169	60.16	60.16	60.33	66.291	66.646	65.959	66.255	66.658	65.928	65.519	66.069	66.274	79.248	80.2	100.0

Table S4. BLASTP matrix (in %) across complete stolbur phytoplasma genomes based on STAMP.

	GOE	PENLEP	POT	c1	c4	c5	o3
GOE	100.000	100.000	41.401	40.252	40.252	41.401	38.650
PENLEP	100.000	100.000	41.401	40.252	40.252	41.401	38.650
POT	41.401	41.401	100.000	89.937	89.937	100.000	89.024
c1	40.252	40.252	89.937	100.000	100.000	89.937	90.854
c4	40.252	40.252	89.937	100.000	100.000	89.937	90.854
c5	41.401	41.401	100.000	89.937	89.937	100.000	89.024
o3	38.650	38.650	89.024	90.854	90.854	89.024	100.000

Table S5. POT secreted proteins (POTS).

Locus tag	Description	Start Position	End Position	Orientation
PSOLA_00590	POT secreted protein 1	54513	54836	-
PSOLA_00650	POT secreted protein 2	56436	57623	-
PSOLA_01050	POT secreted protein 3	110398	111456	+
PSOLA_01070	POT secreted protein 4	113377	114534	+
PSOLA_01110	POT secreted protein 5	118906	119946	+
PSOLA_01640	POT secreted protein 6	181770	183125	-
PSOLA_01670	POT secreted protein 7	184404	184799	+
PSOLA_01720	POT secreted protein 8	188016	188213	+
PSOLA_01980	POT secreted protein 9	221986	222765	+
PSOLA_02530	POT secreted protein 10	264572	265891	+
PSOLA_02560	POT secreted protein 11	271092	271445	+
PSOLA_02590	POT secreted protein 12	273380	273658	+
PSOLA_02700	POT secreted protein 13	280805	281623	-
PSOLA_02790	POT secreted protein 14	294956	295333	-
PSOLA_03340	POT secreted protein 15	353135	354163	+
PSOLA_03520	POT secreted protein 16	374623	375612	+
PSOLA_04380	POT secreted protein 17	469295	470605	+
PSOLA_04410	POT secreted protein 18	474625	475935	+
PSOLA_04730	POT secreted protein 19	509490	509639	+
PSOLA_04850	POT secreted protein 20	519762	520097	-
PSOLA_04910	POT secreted protein 21	524962	525582	-
PSOLA_05200	POT secreted protein 22	546852	547760	-
PSOLA_05330	POT secreted protein 23	556124	556705	-
PSOLA_05500	POT secreted protein 24	573886	574461	-
PSOLA_06010	POT secreted protein 25	628426	629046	+
PSOLA_06470	POT secreted protein 26	672513	673718	+
PSOLA_06490	POT secreted protein 27	674238	675761	
